# The long-term impact of the tricuspid valve intervention after Fontan completion

**DOI:** 10.1186/s43044-025-00701-8

**Published:** 2025-11-05

**Authors:** Yuriy Kulyabin, Ilya Soynov, Timothy Lancaster, Vikram Sood, Jennifer Romano, Richard Ohye, Jiyong Moon

**Affiliations:** 1 Meshalkin National Medical Research Center, Novosibirsk, Russian Federation; 2 University of Michigan Health System, C.S. Mott’s Children’s Hospital, Ann Arbor, USA

**Keywords:** Single ventricle, Fontan procedure, Tricuspid valve, Heart transplantation

## Abstract

**Background:**

The incompetence of the tricuspid valve (TV) may predispose to unfavorable results of Fontan palliation in patients with single right ventricle (RV). This study aims to reveal the effect of TV intervention in patients with single RV on long-term outcomes after Fontan completion.

**Methods:**

A single-center retrospective cohort study was conducted with patients who underwent Fontan completion from 1985 to 2017. There was a total of 678 patients with single RV. A total of 128 patients (18.8%) underwent TV intervention at any stage (TVI group); 30 of them (23.4%) underwent repeat TV surgery (repeat TVI subgroup). The control group comprises 550 patients (81.2%) who had no TV surgery regardless of the degree of TR (non TVI group).

**Results:**

The median follow-up was 8.8 (± 7.6) years. Overall transplant- and Fontan takedown-free survival was 62.5% (95% CI 59.2%-64.9%) at 20 years. The repeat TVI group had significantly lower transplant and takedown-free survival rates (Non TVI 76.5% vs. Single TVI 75.3% vs. repeat TVI 56.0% at 15 years, *P* = 0.02). The younger age at Fontan (1.12 [95% CI 1.02–1.22], *p* = 0.019), repeat TVI (3.33 [95% CI 1.57–7.04], *p* = 0.002), TV intervention after Fontan (6.14 [95% CI 2.60–14.50], *p* < 0.001), significant ventricular dysfunction before Fontan (3.12 [95% CI 1.12–8.30], *p* = 0.028) and any concomitant procedure at Fontan (1.98 [95% CI 1.16–3.37], *p* = 0.013) were the significant risk factors for transplant and takedown free- survival.

**Conclusions:**

Repeat TV intervention during the Fontan was associated with inferior outcomesin patients with morphologic systemic RV. Successful TV intervention could provide comparable long-term survival outcomes to non-TV intervention patients.

## Introduction

In recent eras, the early outcomes of Norwood operation for hypoplastic left heart syndrome (HLHS) and other anatomic variants of single right ventricle (RV) were significantly improved, with an early survival of 88 to 92% in various centers [1–3]. The final stage of univentricular palliation is a Fontan procedure aimed to improve blood oxygenation and decrease preload for single ventricle (SV). However, the late-term post-Fontan outcomes in the single RV remain challenging, with up to 59% of transplant-free survival at 12 years after the Norwood-type procedure [1, 3]. Several risk factors had been outlined for failing Fontan circulation and the incompetence of the tricuspid valve (TV) may predispose to right ventricular dysfunction and is crucial for morphological single RV patients. Significant TV insufficiency has been reported to develop in 37% during the interstage period in single RV and up to 25% of them would receive TV repair during their lifetime [2, 4]. Recent studies suggested that TV repair in patients with morphological single RV positively affects their ventricular function and short-term survival. Patients with developing failing Fontan circulation should undergo surgical reinterventions to improve postoperative outcome and avoid heart transplantation or Fontan takedown. However, the long-term survival effect of the TV repair after Fontan completion remains unclear [4–6].

This study aims to reveal the effect of TV intervention in patients with morphological single RV on long-term outcomes after Fontan completion.

## Methods

A single-center retrospective cohort study was conducted with patients who underwent Fontan completion from 1985 to 2017. Data was obtained by electric and paper chart review and transplant and survival outcomes were linked to the scientific registry of transplant recipients and the national death index. The SRTR (The Scientific Registry of Transplant Recipients) data system includes data on all donors, wait-listed candidates and transplant recipients in the United States, submitted by the members of the Organ Procurement and Transplant Network. The Health Resources and Services Administration, US Department of Health and Human Services, provides oversight to the activities of the Organ Procurement and Transplantation Network and SRTR contractors. The University of Michigan Institutional Review Board exempted the approval of this study (HUM 00245866, date 12/5/2023) and the need for informed consent was waived. The TV and ventricular function data were obtained by echocardiogram, performed before and after Fontan completion, at the time of patient discharge, and 3,5,10, 15 and 20 years after Fontan. This study excluded interstage mortality and failed staged palliation, therefore, the part of patients who needed TV intervention during the interstage period would be excluded from this study. Follow-up data was available for 87% patients, because many patients received post-Fontan care elsewhere. However, survival and transplant follow-up data were obtained from our electronic medical records, the National Death Index and SRTR.

The cohort we analyzed consists of 487 patients with HLHS, 66 patients with heterotaxy syndrome and 125 patients with different anatomical variations with single RV. Patients with common AV valve were excluded.

Patients underwent TV intervention were included into TVI group. The non TVI group comprises patients who had not received TV surgery. Those patients who underwent repeat TV surgery were included in the repeat TVI subgroup for further analysis.

We indicated a transplant and Fontan takedown-free survival as the primary outcome. Subgroup analysis was performed for the repeat TVI group. Risk factor analysis of the primary outcome was performed. In the risk analysis, concomitant procedures at the time of Fontan included not only TV repair or replacement but also branch pulmonary artery augmentation, superior vena cava augmentation, pacemaker implantation, Maze procedure, re-coarctation repair, sub aortic stenosis repair.

Tricuspid regurgitation was characterized on the grading scale and was defined as follows: 0, none; 1+, mild (narrow regurgitant jet at the orifice [< 2 mm], single jet); 2+, mild to moderate (wider jet area at orifice [< 4 mm], multiple jets, or both, mild atrial enlargement); 3+, moderate to severe (wide jet orifice, jet reaches back wall of atrium, moderate atrial enlargement); and 4+, severe (wide jet orifice, jet reaches back wall of atrium, reversal of flow in the pulmonary or hepatic veins, severe atrial enlargement). A successful outcome was defined as 0 to 2 + TR. TV intervention was undertaken for the presence of 3 to 4 + TR, regardless of symptoms.

### Statistical analysis

Statistical analysis was performed using Stata 17 (StataCorp MP, College Station, TX, USA) and statistical significance was assessed at the p-value < 0.05. Descriptive statistics are presented as counts and percentages for categorical variables and medians with interquartile range (25th– 75th percentiles) for continuous data. Time-dependent outcomes (takedown, death, or transplantation) were analyzed using survival analysis methods. Kaplan–Meier plots were constructed for each event of interest and stratified by clinical subgroups. The Wilcoxon test was used to compare survival distributions among patient subgroups.

### Operative technique

A multidisciplinary decision-making process determined the indication of the TV repair according to the mechanism of TV incompetence. The saline test was performed to determine the cause of the incompetence. Intraoperative determination was made of the zone of regurgitation jet, the diameter of the annulus, leaflet prolapse and leaflet tethering. Various techniques were utilized to address the TV insufficiency according to the TV function and anatomy such techniques included annuloplasty, leaflet mobilization and chordal detachment. Partial annuloplasty with running parallel mattress sutures from the anteroposterior commissure to the posteroseptal commissure was commonly applied for patients with anterior leaflet prolapse or annular [6]. Commissuroplasty and clefts closure were used in localized areas of leaflet prolapse. Patient might have undergone more than one repair method to achieve satisfying valve function at any stage of the palliative pathway. Patients who underwent unsuccessful TV reconstruction required TV replacement with further repeat TV interventions.

## Results

### Baseline characteristics

The baseline characteristics of the cohorts were presented in Table 1. The median follow-up was 8.8 (± 7.6) years. Among the 128 TV intervention patients, 30 underwent repeat TV intervention (23%). Fifty-five (43%) patients underwent the initial TV repair before Fontan (40 patients—at stage 2, 15 patients —during the interstage period), 60 (47%) patients at the time of Fontan and 13 (10%) patients after Fontan completion.

**Table 1:** Baseline characteristics of the cohort

Characteristic	Non TVi group (*n*-550)	TVI group (*n*-128)	Repeat TVI subgroup (*n*-30)	*P* (non TVI and TVI)	Subgroup *P* (non TVI and repeat TVI)
Sex					
Male	360 (65.45%)	78 (60.9%)	17 (56.7%)		
Female	190 (34.55%)	50 (39.1%)	13 (43.3%)	0.33	0.33
HLHS, n	396 (72%)	91 (71.1%)	21 (70%)	0.83	0.83
Heterotaxy, n	49 (8.91%)	17 (13.3%)	5 (16.7%)	0.13	0.18
Hemifontan, n	519 (94.36%)	124 (96.9%)	29 (96.7%)	0.24	1.00
PAP > 15 before Fontan, n	90 (16.36.9%)	24 (18.7%)	6 (20%)	0.50	0.60
EDP > 10 before Fontan, n	160 (29.09%)	43 (33.6%)	14 (46.7%)	0.31	**0.039**
Any procedure at the time of Fontan, n	66 (12%)	83 (64.8%)	21 (70%)	**< 0.001**	**< 0.001**
Age at Fontan, years	2.03 (1.7; 2.69)	2.14 (1.8; 3.0)	2.2 (1.9; 3.1)		
Era					
Before 1998	156 (28.36%)	33 (25.8%)	10 (33.3%)		
1998–2008	233 (42.36%)	62 (48.4%)	15 (50%)	0.07	0.13
After 2008	161 (29.27%)	33 (25.8%)	5 (16.7%)	0.45	0.34

*TVI* tricuspid valve intervention,* HLHS* hypoplastic left heart syndrome,* PAP* pulmonary artery pressure,* EDP* end-diastolic pressure. Significant values are bolded

The non TVI and TVI groups had no significant differences in background characteristics except for concomitant surgical procedures at the time of Fontan (non TVI 12% vs. TVI 64.8%, *p* < 0.001). Sixty patients (47%) underwent TV reconstruction. Other concomitant procedures included pulmonary artery augmentation, arch reintervention, neoaortic valve intervention. Patients in the non TVI group had an equal rate of significant ventricular dysfunction at the time of Fontan compared with the TVI group (no TVI 2.5% vs. TVI 3.9%, *p* = 0.40) (Table 2). There were no significant differences in early mortality during the Fontan completion between the groups (non TVI 4.9% vs. TVI 6.2%, *p* = 0.61) nor the subgroup (repeat TVI 6.6% vs. non TVI 4.9%, *p* = 0.27).

**Table 2:** Results after Fontan completion

Characteristics	Non TVI group (*n*-550)	TVI group (*n*-128)	Repeat TVI subgroup (*n*-30)	*P* (non TVI and TVI)	Subgroup *P* (non TVI and repeat TVI)
Significant SV dysfunction before Fontan, n	14 (2.55%)	5 (3.9%)	1 (3.3%)	0.40	0.55
Prolonged chest drains (> 2weeks), n	129 (23.45%)	33 (25.8%)	7 (23.3%)	0.62	1.00
Early failure (Fontan takedown), n	33 (6%)	11 (8.5%)	3 (10%)	0.55	0.77
Early mortality, n	27 (4.9%)	8 (6.2%)	2 (6.6%)	0.61	0.27
Length of hospital stay, days	12 (8.5; 18)	11 (8; 16)	10 (8; 17)	0.62	0.77
Overall mortality, n	105 (19.2%)	31 (24.3%)	10 (32.4%)	0.46	0.27
Significant SV dysfunction after Fontan, n	68 (13%)	20 (16.6%)	8 (28.5%)	0.36	**0.012**
Primary outcome at 15 years, n	129 (23.5%)	36 (28.5%)	14 (49.4%)	0.061	**0.002**

*TVI* tricuspid valve intervention,* SV* single ventricle. Significant values are bolded

### Primary outcome

Sixty-eight survivors (13%) in the non TVI group had significant SV dysfunction after Fontan completion, most of them had 3 + TR grade (41 patients). Twenty survivors (16.6%) in the TVI group had significant SV dysfunction after Fontan completion (*p* = 0.36).

There were no significant differences in mortality during the study period after Fontan between the groups (non TVI 19.2% vs. TVI 24.3%, *p* = 0.46) nor the subgroup (repeat TVI 32.4% vs. non TVI 19.2%, *p* = 0.27).

**Fig. 1:**
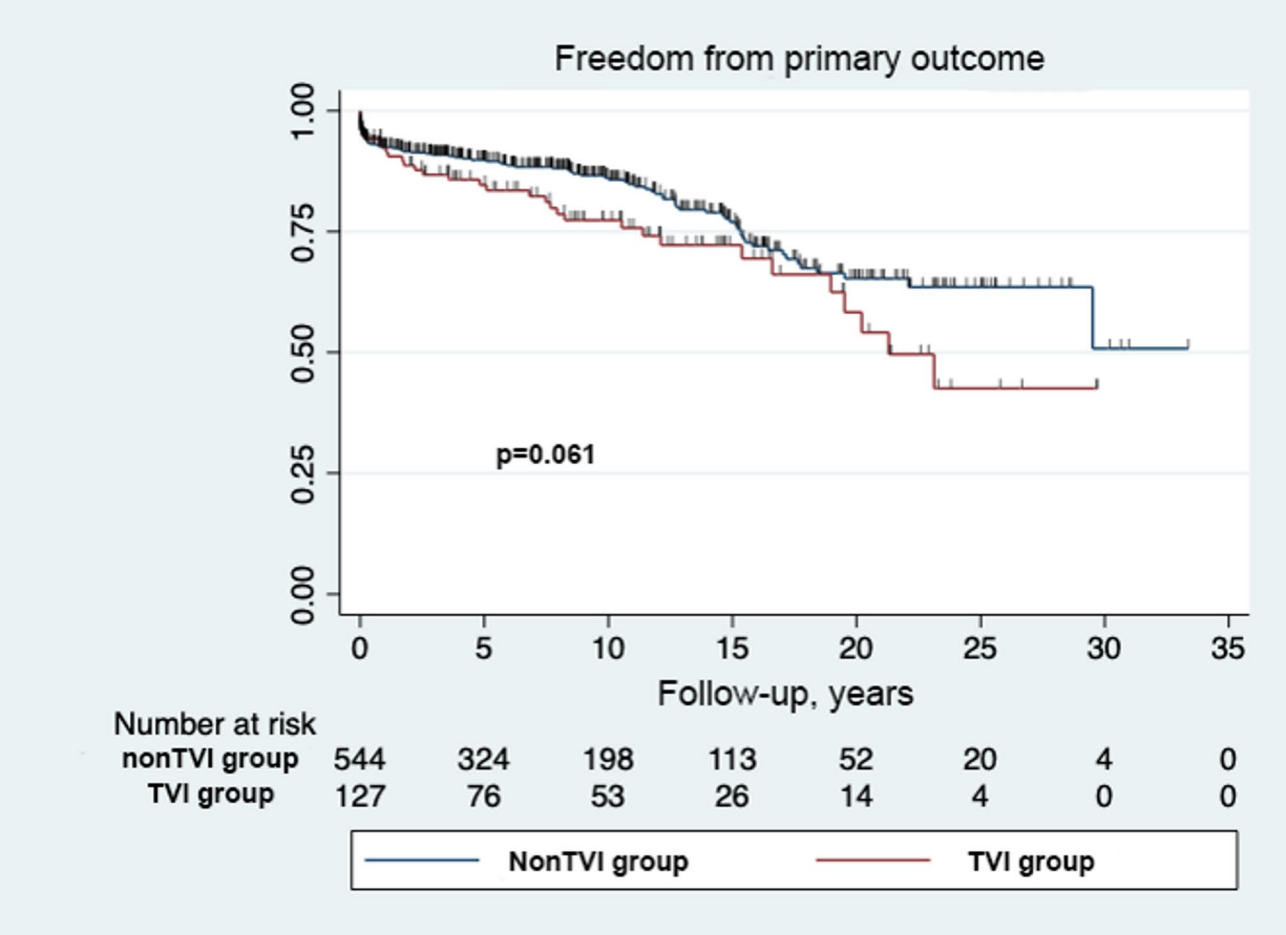
– Kaplan-Meier curve of freedom from unfavorable outcome after Fontan in patients from TVI and non TVI groups. TVI-tricuspid valve intervention; non TVI – no tricuspid valve intervention

**Fig. 2:**
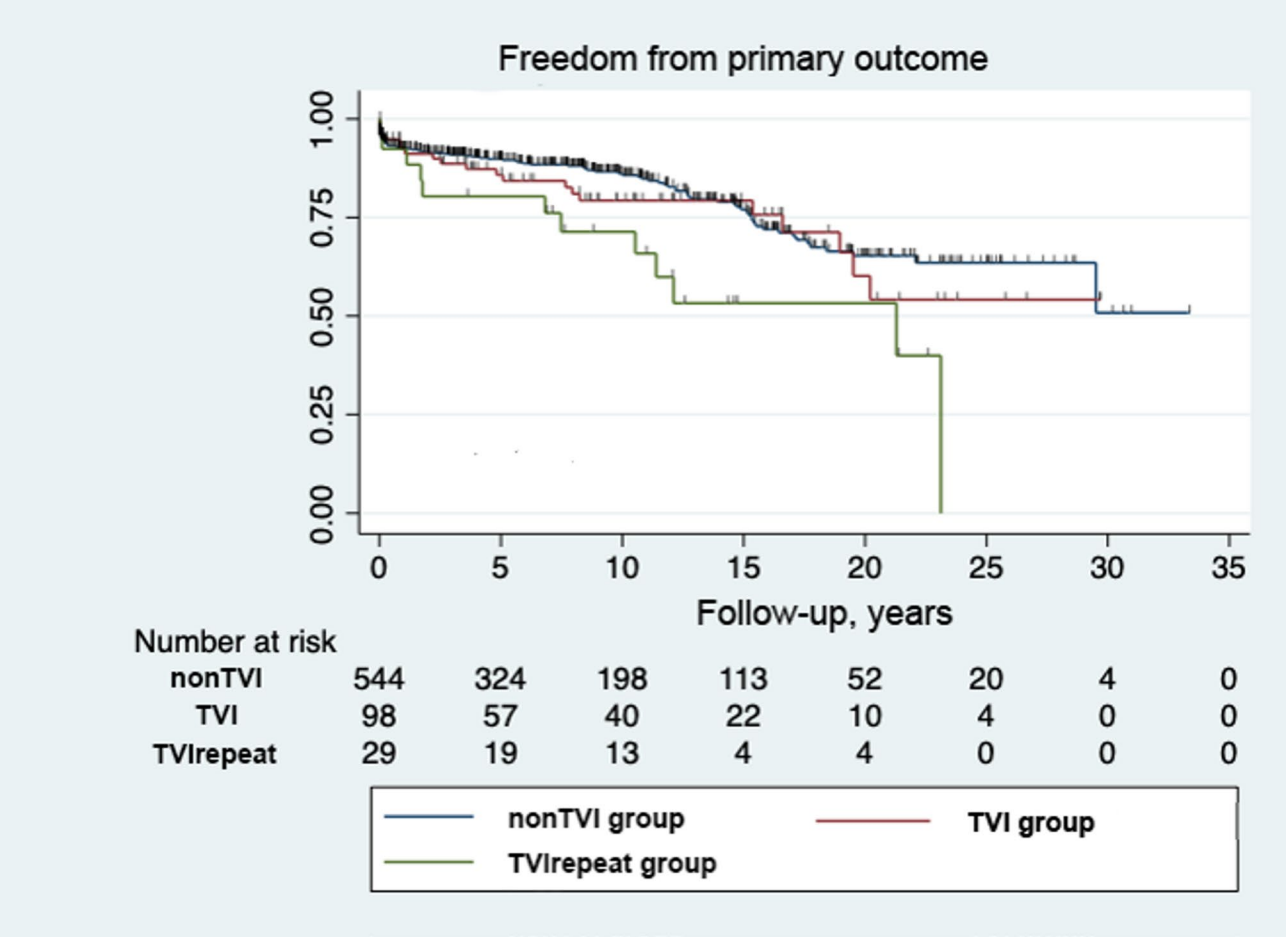
- Kaplan-Meier curve of freedom from unfavorable outcome after Fontan including the patients from repeat TVI subgroup. TVI-tricuspid valve intervention; non TVI – no tricuspid valve intervention; TVI repeat – any repeat tricuspid valve intervention

Transplant- and Fontan takedown-free survival was 62.5% (95% CI 59.2%-64.9%) at 20 years (Fig. 1). Although the TVI group tended to have lower conditional survival than the non TVI group, there was no statistical difference at 15 years (non TVI 23.5% [95% CI 21.2%-25.6%] vs. TVI 28.5% [95% CI 26.4%-29.8%], *P* = 0.061). However, the subgroup analysis revealed that the repeat TVI group had significantly lower survival rates compared to non TVI or single TVI groups (Non TVI 76.5% vs. Single TVI 75.3% vs. repeat TVI 56.0% at 15 years, *P* = 0.02) (Fig. 2).

### Follow-up echocardiography data

Ninety-eight patients from the TVI group without repeat TV plasty and 30 patients who underwent repeat TVI had available echo data in the follow-up. Only 490 patients from the non TVI group (89%) had available echo data in the follow-up. Thirty-four patients (35%) in TVI group and 14 (50%) patients in repeat TVI group had more than moderate TV regurgitation. Forty-one patients (8.4%) from the non TVI group were observed with 3 + TV regurgitation grade (Table 3).

**Table 3:** Echocardiography AVVR follow-up data among TVI group

AVVR	TVI group (*n*-98)	Repeat TVI group (*n*-30)	Non TVI group (*n*-490)
Echo follow-up, years	6.79 (± 7.85)	6.34 (± 5.56)	8.8 (± 7.6)
None, n	17 (19.3%)	5 (19.2%)	285 (58.1%)
2+, n	37 (42%)	7 (26.9%)	164 (33.5%)
3+, n	28 (31.8)	10 (38.5%)	41 (8.4%)
4+, n	6 (6.8%)	4 (15.4%)	none

*AVVR* atrioventricular valve regurgitation, * TVI* tricuspid valve intervention

### Risk factor analysis

Risk factor analysis revealed that the younger age at Fontan (1.12 [95% CI 1.02; 1.22], *p* = 0.019), repeat TV surgery (3.33 [95% CI 1.57; 7.04], *p* = 0.002), TV intervention after Fontan completion (6.14 [95% CI 2.60; 14.50], *p* < 0.001), significant systemic single ventricular dysfunction at Fontan (3.12 [95% CI 1.12; 8.30], *p* = 0.028) and any concomitant procedure at the time of Fontan (1.98 [95% CI 1.16; 3.37], *p* = 0.013) were the significant risk factors for transplant- and Fontan takedown-free survival (Tables 4 and 5).

**Table 4:** Univariate analysis for primary outcome

Parameter	Univariate	*p*
Age at Fontan	**1.12 [1.02; 1.22]**	**0.010**
repeat TV surgery	**3.33 [1.57; 7.04]**	**0.002**
TVI after Fontan	**6.14 [2.60; 14.50]**	**< 0.001**
SV dysfunction before Fontan	**3.15 [1.24; 8.00]**	**0.016**
Moderate AVVR before Fontan	1.38 [0.81; 2.33]	0.22
Anatomical subtypes of single RV	0.97 [0.53; 1.76]	0.14
Any concomitant procedure at the time of Fontan	**1.80 [1.14; 2.83]**	**0.011**
Surgical era	**0.37 [0.27; 0.50]**	**< 0.001**

*TV* tricuspid valve, * TVI* tricuspid valve intervention,* SV* single ventricle, * AVVR* atrioventricular valve regurgitation, * RV* right ventricle. Significant values are bolded

**Table 5:** Multivariate analysis for primary outcome

parameter	Multivariate	*p*
Age at Fontan	1.11 [1.01; 1.22]	**0.019**
repeat TV surgery	1.54 [0.57; 4.13]	0.38
TVI after Fontan	2.53 [0.94; 6.83]	0.066
SV dysfunction before Fontan	3.12 [1.12; 8.30]	**0.028**
Moderate AVVR before Fontan	0.97 [0.53; 1.76]	0.93
Any concomitant procedure at the time of Fontan	1.98 [1.16; 3.37]	**0.013**
Surgical era	0.38 [0.28; 0.52]	**< 0.001**

*TV* tricuspid valve,* TVI* tricuspid valve intervention,* SV* single ventricle,* AVVR* atrioventricular valve regurgitation. Significant values are bolded

## Discussion

The survival rate after Fontan completion has been significantly improved during the past decade [7–9]. However, up to half of the patients with HLHS developed a failing Fontan circulation and required transplantation [1]. Several risk factors in the early postoperative period had been outlined for failing Fontan circulation, such as low cardiac output, elevated pulmonary artery pressure and prolonged pleural effusion [10]. Despite the etiology and clinical signs, patients with developing failing Fontan circulation should undergo aggressive medical therapy and surgical reinterventions to improve postoperative outcome and avoid heart transplantation or Fontan takedown.

In patients with single RV, TV insufficiency should be considered as the most likely cause of significant ventricular dysfunction. More than 30% of patients with HLHS have any significant degree of TR. The etiology is commonly multifactorial and is still not fully understood, as some neonates have TR early after birth [2]. It is believed that the RV changes its shape and geometry after birth with increased volume load, which impairs the orientation of the papillary muscles and dilates the fibrous ring [2, 5]. TR further worsens the ventricular function with volume overload that starts the pathophysiological vicious cycle. Other causes of TR included structural anomalies of the subvalvular apparatus and leaflets dysplasia [5, 11]. Alsoufi et al. showed that only half of the patients who underwent TV repair had annular dilatation, while others had various structural abnormalities [2]. The group from Boston emphasized that the most common preoperative mechanism of TR they had was annular dilatation with anterior leaflet prolapse associated with poor outcomes and despite the early successful result of TV repair, the long-term ability of TV to support the systemic RV is limited [5]. Our study’s most common TV repair technique was annuloplasty with partial obliteration of the posterior leaflet, however, it was mostly used in combination with other techniques according to the valve morphology. Unfortunately, we were not able to analyze the mechanism of TR in the current cohort in detail. Previously, we had proven this technique to be the most effective in improving the TV function in patients with HLHS in short term [3, 6].

In our study, 8.4% of patients did not undergo any TVI before Fontan despite moderate TR. These patients had a significant ventricular dysfunction at the time of Fontan completion (13%) with poor outcome. However, the early postoperative course and late-term outcomes were comparable with both non TVI and TVI groups. On the other hand, all patients in the TVI group had moderate-to-severe TR and only 3.9% of them had ventricular dysfunction. This may indicate that besides structural TV anomalies and RV dilatation, TR may have other significant factors, such as RV desynchrony, age and body weight during the Norwood stage [11–13]. The second stage of palliative repair aims to unload the ventricle and usually improves both TV and ventricular function. Ashburn et al. showed that patients who underwent TV repair at the time of the Norwood or the second stage palliation have a high risk of TR recurrence and late mortality [14]. We also have found that early age, repeat TV interventions and TV repair after Fontan completion are significant risk factors for unfavorable long-term outcomes. Perhaps, the early unfavorable TV repair due to structural anomalies predisposes to the further development of RV dysfunction that worsening the TR in the long-term period.

In our study, a third of the patients underwent initial TV repair at the second stage of palliation and about half underwent redo repair after the initial repair. In contrast, patients who underwent TV repair during or after Fontan did not require further reinterventions. In our opinion, the satisfying TV function should be achieved as early as possible, especially it feels crucial for the patients who had unsuccessful initial TV repair. Repeat TVI often requires more complex repair to restore the TV function and it takes longer bypass time that also may reflects on the SV function. Alsoufi et al. concluded that TV repair could be successfully performed in most patients. However, the rate of TR recurrence with worsening of the ventricular function is still high and associated with poor outcomes [2]. We found no significant conditional survival differences between the TVI and non TVI groups, but patients who received repeat TV intervention had significantly lower transplant and Fontan takedown-free survival (56%). Patients who required repeat TVI had higher rate of significant RV dysfunction (28.5%). Successful TV repair should be achieved as early as possible at the any stage, but it also is better to make it before severe ventricular dysfunction. However, the meticulous assessment of the TV function could be the key to improving ventricular function, a recurrent TR should be considered a sign of severely progressive RV dysfunction. It is the cornerstone to define what is the reason and what is the consequence. In presence of severe ventricular dysfunction and history of unsuccessful TV repair, the one more intervention may have harmful effect even if the TV function restored. Patients with a single RV and significant TR should be assessed individually for TV surgery, closely evaluating the mechanism of TR and ventricular function.

## Conclusion

Repeat TV intervention during the single ventricle palliation was associated with inferior transplant and Fontan-takedown free survival in patients with morphologic systemic right ventricle. Successful TV intervention at the any palliative stage could provide comparable long-term survival outcomes to non-TV intervention patients after Fontan completion.

## Limitations

In this retrospective cohort study, one of the limitations was the absence of detailed echocardiography data, which restricted us from assessing the TR mechanism in patients. Another limitation was incomplete follow-up (follow-up data was available for 87% patients) because many patients live remotely from our center and receive post-Fontan care elsewhere. However, survival and transplant follow-up data were obtained from our electronic medical records, the National Death Index and SRTR. This study excluded inter-stage mortality and failed staged palliation. Therefore, the part of patients who needed TV intervention during the interstage would be excluded from this study. The selection bias would be one of the limitations of this study.

## Data Availability

No datasets were generated or analysed during the current study.

## Abbreviations

HLHS: Hypoplastic left hear syndrome
RV: Right ventricle
TV: Tricuspid valve
TVI: Tricuspid valve intervention
TR: Tricuspid regurgitation
